# Supplementary material for: Modeling glioblastoma heterogeneity as a dynamic network of cell states
Source: Mol Syst Biol. 2021 Sep 16;17(9):e10105. doi: 10.15252/msb.202010105 (PMC8444284; doi:10.15252/msb.202010105)
Supplement: Supplementary file 6 — Source Data for Figure 5 [file MSB-17-e10105-s004.zip › Figure5A_sourcedata/GSEA_3017/nosigdb_stateD.GseaPreranked.1622795010397/RICHARDS_NOWAKOWSKI_SCIENCE_2017_IPC_NEN3_UPREG.html]

Details for gene set RICHARDS\_NOWAKOWSKI\_SCIENCE\_2017\_IPC\_NEN3\_UPREG[GSEA]

|  || Dataset | state23017 |
| Phenotype | NoPhenotypeAvailable |
| Upregulated in class | na\_pos |
| GeneSet | RICHARDS\_NOWAKOWSKI\_SCIENCE\_2017\_IPC\_NEN3\_UPREG |
| Enrichment Score (ES) | 0.32490882 |
| Normalized Enrichment Score (NES) | 2.8752959 |
| Nominal p-value | 0.0 |
| FDR q-value | 0.0 |
| FWER p-Value | 0.0 |
Table: GSEA Results Summary

  

Fig 1: Enrichment plot: RICHARDS\_NOWAKOWSKI\_SCIENCE\_2017\_IPC\_NEN3\_UPREG      
 Profile of the Running ES Score & Positions of GeneSet Members on the Rank Ordered List

  

| PROBE | GENE SYMBOL | GENE\_TITLE | RANK IN GENE LIST | RANK METRIC SCORE | RUNNING ES | CORE ENRICHMENT || 1 | FABP5 |  |  | 1 | 0.885 | 0.0445 | Yes |
| 2 | RPS24 |  |  | 7 | 0.568 | 0.0696 | Yes |
| 3 | FJX1 |  |  | 12 | 0.514 | 0.0927 | Yes |
| 4 | MEX3A |  |  | 30 | 0.402 | 0.0998 | Yes |
| 5 | RPL13A |  |  | 36 | 0.381 | 0.1154 | Yes |
| 6 | RPL23 |  |  | 51 | 0.349 | 0.1221 | Yes |
| 7 | RPL10 |  |  | 53 | 0.344 | 0.1389 | Yes |
| 8 | RPS8 |  |  | 54 | 0.343 | 0.1565 | Yes |
| 9 | RPL12 |  |  | 56 | 0.342 | 0.1732 | Yes |
| 10 | HES6 |  |  | 60 | 0.336 | 0.1880 | Yes |
| 11 | RPL28 |  |  | 62 | 0.330 | 0.2041 | Yes |
| 12 | EIF3A |  |  | 79 | 0.312 | 0.2074 | Yes |
| 13 | ZEB1 |  |  | 84 | 0.303 | 0.2198 | Yes |
| 14 | RPL18 |  |  | 87 | 0.302 | 0.2336 | Yes |
| 15 | MARCKS |  |  | 96 | 0.295 | 0.2424 | Yes |
| 16 | GDI2 |  |  | 107 | 0.285 | 0.2490 | Yes |
| 17 | RPLP0 |  |  | 108 | 0.285 | 0.2636 | Yes |
| 18 | CHD7 |  |  | 116 | 0.278 | 0.2723 | Yes |
| 19 | TMEM158 |  |  | 120 | 0.275 | 0.2840 | Yes |
| 20 | RPS27A |  |  | 125 | 0.273 | 0.2948 | Yes |
| 21 | RPS25 |  |  | 133 | 0.268 | 0.3030 | Yes |
| 22 | RPL35A |  |  | 139 | 0.263 | 0.3125 | Yes |
| 23 | RPL8 |  |  | 155 | 0.254 | 0.3136 | Yes |
| 24 | RPS16 |  |  | 158 | 0.251 | 0.3249 | Yes |
| 25 | POU2F1 |  |  | 194 | -0.253 | 0.3102 | No |
| 26 | RFTN2 |  |  | 219 | -0.255 | 0.3043 | No |
| 27 | CELSR2 |  |  | 220 | -0.255 | 0.3174 | No |
| 28 | ARID1A |  |  | 335 | -0.267 | 0.2410 | No |
| 29 | MAP6 |  |  | 369 | -0.270 | 0.2287 | No |
| 30 | RPN2 |  |  | 376 | -0.271 | 0.2378 | No |
| 31 | OXR1 |  |  | 388 | -0.272 | 0.2431 | No |
| 32 | MED27 |  |  | 397 | -0.273 | 0.2507 | No |
| 33 | TMTC2 |  |  | 399 | -0.273 | 0.2639 | No |
| 34 | COL4A5 |  |  | 470 | -0.282 | 0.2230 | No |
| 35 | SNCAIP |  |  | 540 | -0.290 | 0.1833 | No |
| 36 | HDAC9 |  |  | 548 | -0.291 | 0.1927 | No |
| 37 | LZTS1 |  |  | 564 | -0.293 | 0.1958 | No |
| 38 | NEDD4L |  |  | 595 | -0.299 | 0.1874 | No |
| 39 | PAX6 |  |  | 629 | -0.307 | 0.1770 | No |
| 40 | ZHX3 |  |  | 642 | -0.309 | 0.1833 | No |
| 41 | ENC1 |  |  | 643 | -0.309 | 0.1991 | No |
| 42 | ZFHX4 |  |  | 656 | -0.311 | 0.2055 | No |
| 43 | MLC1 |  |  | 701 | -0.320 | 0.1871 | No |
| 44 | PON2 |  |  | 738 | -0.328 | 0.1755 | No |
| 45 | TMSB4X |  |  | 742 | -0.329 | 0.1899 | No |
| 46 | MEGF10 |  |  | 790 | -0.341 | 0.1702 | No |
| 47 | NKAIN3 |  |  | 828 | -0.351 | 0.1590 | No |
| 48 | JAKMIP2 |  |  | 837 | -0.354 | 0.1708 | No |
| 49 | GLUL |  |  | 920 | -0.382 | 0.1255 | No |
| 50 | SETBP1 |  |  | 1106 | -0.471 | 0.0035 | No |
| 51 | MDM2 |  |  | 1135 | -0.499 | 0.0069 | No |
| 52 | DAAM1 |  |  | 1161 | -0.523 | 0.0139 | No |
| 53 | SDC2 |  |  | 1200 | -0.580 | 0.0136 | No |
| 54 | SLC1A3 |  |  | 1259 | -0.728 | 0.0050 | No |
| 55 | SYNE2 |  |  | 1282 | -0.829 | 0.0300 | No |
Table: GSEA details [plain text format]

  

Fig 2: RICHARDS\_NOWAKOWSKI\_SCIENCE\_2017\_IPC\_NEN3\_UPREG: Random ES distribution      
 Gene set null distribution of ES for **RICHARDS\_NOWAKOWSKI\_SCIENCE\_2017\_IPC\_NEN3\_UPREG**
